# Low-frequency transcranial stimulation of pre-supplementary motor area alleviates levodopa-induced dyskinesia in Parkinson’s disease: a randomized cross-over trial

**DOI:** 10.1093/braincomms/fcaa147

**Published:** 2020-09-18

**Authors:** Allan Lohse, David Meder, Silas Nielsen, Anders Elkjær Lund, Damian M Herz, Annemette Løkkegaard, Hartwig R Siebner

**Affiliations:** f1 Danish Research Centre for Magnetic Resonance, Centre for Functional and Diagnostic Imaging and Research, Copenhagen University Hospital Hvidovre, Hvidovre 2650, Denmark; f2 Department of Neurology, Copenhagen University Hospital Bispebjerg, Copenhagen 2400, Denmark; f3 Faculty of Medical and Health Sciences, Institute for Clinical Medicine, University of Copenhagen, Copenhagen 2200, Denmark

**Keywords:** functional MRI, levodopa-induced dyskinesia, pre-supplementary motor area, repetitive transcranial magnetic stimulation

## Abstract

Levodopa-induced dyskinesia gradually emerges during long-term dopamine therapy, causing major disability in patients with Parkinson disease. Using pharmacodynamic functional MRI, we have previously shown that the intake of levodopa triggers an excessive activation of the pre-supplementary motor area in Parkinson disease patients with peak-of-dose dyskinesia. In this pre-registered, interventional study, we tested whether the abnormal responsiveness of the pre-supplementary motor area to levodopa may constitute a ‘stimulation target’ for treating dyskinesia. A gender-balanced group of 17 Parkinson disease patients with peak-of-dose dyskinesia received 30 min of robot-assisted repetitive transcranial magnetic stimulation, after they had paused their anti-Parkinson medication. Real-repetitive transcranial magnetic stimulation at 100% or sham-repetitive transcranial magnetic stimulation at 30% of individual resting corticomotor threshold of left first dorsal interosseous muscle was applied on separate days in counterbalanced order. Following repetitive transcranial magnetic stimulation, patients took 200 mg of oral levodopa and underwent functional MRI to map brain activity, while they performed the same go/no-go task as in our previous study. Blinded video assessment revealed that real-repetitive transcranial magnetic stimulation delayed the onset of dyskinesia and reduced its severity relative to sham-repetitive transcranial magnetic stimulation. Individual improvement in dyskinesia severity scaled linearly with the modulatory effect of real-repetitive transcranial magnetic stimulation on task-related activation in the pre-supplementary motor area. Stimulation-induced delay in dyskinesia onset correlated positively with the induced electrical field strength in the pre-supplementary motor area. Our results provide converging evidence that the levodopa-triggered increase in pre-supplementary motor area activity plays a causal role in the pathophysiology of peak-of-dose dyskinesia and constitutes a promising cortical target for brain stimulation therapy.

## Teaser

Levodopa-induced dyskinesia constitutes a major side-effect to dopamine treatment of Parkinson’s disease. Lohse *et al.* show that low-frequency transcranial magnetic stimulation of the pre-supplementary motor area alleviates levodopa-induced dyskinesia. A causal contribution of pre-supplementary motor area in the pathophysiology of levodopa-induced dyskinesia is indicated and pre-supplementary motor area is identified as a candidate target for future stimulation-based therapy.

## Graphical Abstract

**Graphical Abstract:**
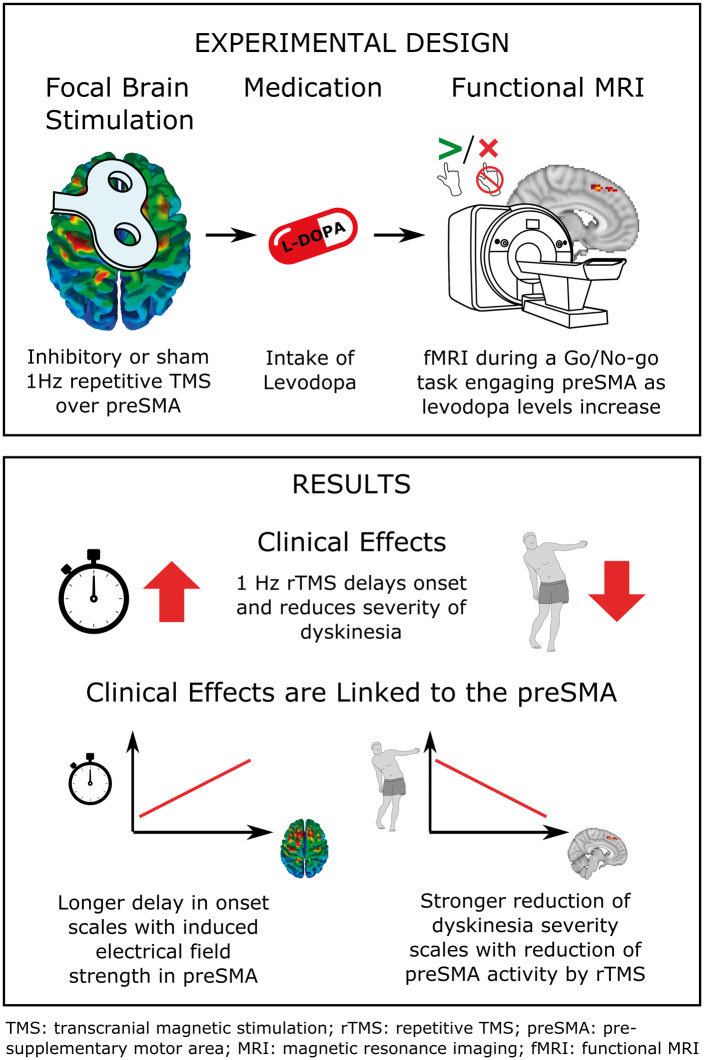

## Introduction

Progressive nigrostriatal dopaminergic denervation causes the cardinal motor symptoms of Parkinson disease (Postuma *et al.*, 2015). The striatal dopaminergic deficit can be compensated by dopamine replacement therapy. Chronic pharmacological treatment gradually leads to abnormal involuntary movements known as levodopa-induced dyskinesia (LID; Ahlskog and Muenter, 2001; Ha and Jankovic, 2011). The most common clinical manifestation is peak-of-dose LID occurring when the brain dopamine concentration peaks (Ha and Jankovic, 2011). Molecular imaging in humans as well as research in animals has identified maladaptive plasticity of the cortico-striatal synapses in the motor territories of the basal ganglia as a key pathophysiological mechanism involving multiple neurotransmitters (Pavese *et al.*, 2006; Picconi *et al.*, 2008; Rylander *et al.*, 2010; Nishijima *et al.*, 2014). While the ‘classic’ model assumes that an imbalance of the direct (D1 receptor) and indirect (D2 receptor) pathways in the motor cortico-basal ganglia loops plays a central role in the aetiology of LID (Cenci and Konradi, 2010; Zhai *et al.*, 2019), more recent studies suggest that a dysfunction of limbic and associative non-motor circuits may contribute as well (Barroso-Chinea and Bezard, 2010; Espay *et al.*, 2018; Donzuso *et al.*, 2020).

To study the pathophysiology of LID at the brain circuit level, we had previously developed a pharmacological functional magnetic resonance imaging (fMRI) approach that probes the functional impact of levodopa on the motor network in the period from levodopa intake to the onset of LID (Herz *et al.*, 2014). We showed that the intake of levodopa triggers an excessive functional response of the pre-supplementary motor area (preSMA) and putamen in Parkinson’s disease patients with LID compared to patients without LID (Herz *et al.*, 2014). This excessive activity in preSMA was present when patients had to withhold a motor response during a go/no-go task. The activation peaked in the rostral SMA scaled with the individual severity of LID (Herz *et al.*, 2014). Additional analyses of resting-state functional connectivity and task-related effective connectivity showed alterations in functional connectivity among these areas in LID patients (Herz *et al.*, 2015, 2016). These results implicate the preSMA in the pathophysiology of LID, but due to the correlational nature of fMRI, any mechanistic interpretation remains ambiguous. On one hand, excessive preSMA activation may reflect a compensatory mechanism that suppresses a levodopa-induced activation of the hypersensitized striatum. On the other hand, it may be part of the maladaptive process itself, contributing to dyskinesia.

In this study, we adopted an interventional approach to overcome this ambiguity and to probe the causal role of preSMA in LID. We applied inhibitory 1-Hz repetitive transcranial magnetic stimulation (rTMS) to suppress neuronal activity within the preSMA and tested the acute effects of preSMA stimulation on the onset and severity of LID. Using robot-assisted rTMS under stereotactic guidance, we precisely targeted the coordinate of peak preSMA activation as revealed by our previous fMRI study (Herz *et al.*, 2014). We chose 1-Hz rTMS as interventional protocol, because previous studies had reported some beneficial effect of inhibitory 1-Hz rTMS of the SMA on LID (Koch *et al.*, 2005; Brusa *et al.*, 2006; Sayın *et al.*, 2014). Assuming a maladaptive role of the excessive preSMA response to levodopa, we hypothesized that real 1-Hz rTMS over the preSMA would delay the time from levodopa intake to LID onset and reduce LID severity relative to control rTMS. Using the same task-based fMRI paradigm as in our previous study (Herz *et al.*, 2014), we tested whether the individual post-rTMS reduction in preSMA activity scaled with clinical improvement of LID. Informed by structural MRIs of the individual brain, we modelled the distribution of the induced electrical field in each patient. This enabled us to test whether the local electrical field strength induced by real 1-Hz rTMS in the preSMA is predictive of the beneficial effects of preSMA stimulation on LID expression.

## Materials and methods

### Patients

Twenty patients with a clinical diagnosis of Parkinson’s disease (Hoehn & Yahr Stages 1–3) and clinical documentation of peak-of-dose dyskinesia were enrolled in the study. Seventeen patients were included in the final analysis. Their clinical and demographic characteristics are listed in Table 1. Three patients had to be excluded from the study. One patient turned out to have diphasic rather than peak-of-dose dyskinesias and two patients completed only the first experimental session. The lack of comparable studies precluded power calculations for our two primary outcome measures, namely the rTMS-induced change in the levodopa-induced rise in preSMA activation during NoGo trials and the change in LID onset and severity. Therefore, we based our sample size estimation on two considerations. First, we had been able to demonstrate a between-group difference in the brain’s functional response to levodopa in 13 patients with and 13 patients without LID (Herz *et al.*, 2014, 2015). Second, 10 single-session inhibitory rTMS studies had been published until 2016, including 2–20 patients (median = 10) showing positive effects on dyskinesia (Table 2). Given this previous knowledge and assuming a drop-out rate of maximal 20%, we reasoned that 20 patients would secure sufficient statistical power for our two primary outcome measures. Patients were studied in an OFF-medication state after withdrawal from their usual dopamine replacement therapy, i.e. 12 h for levodopa and 48 h for dopamine agonists, MAO-B and COMT inhibitors. Exclusion criteria were insufficient Danish language skills, neurological disease other than Parkinson’s disease, major psychiatric illness, sedatives or serotonergic medication as current treatment, severe tremor, Montreal Cognitive Assessment score < 26 as well as contraindication for transcranial magnetic stimulation, including epilepsy or epilepsy in first degree relatives, and for MRI, i.e. pacemaker, pregnancy, metallic foreign bodies inside the body and severe claustrophobia. All participants gave their informed consent in accordance with the Declaration of Helsinki. The study was approved by the regional ethics committee (H-15017863) and the design and main hypotheses of the study were pre-registered on clinicaltrials.gov ahead of data acquisition (NCT03354455).

**Table 1:** Overview of clinical and demographic characteristics

Variable	*N* = 17
Sex	8 F
Handedness	16 R
Age, years	67.8 ± 7.8
Education, years	14.3 ± 2.7
MoCA	27.5 ± 1.7
GDS-30	5.1 ± 4.6
BIS-11	57.7 ± 6.3
Disease duration, years	12.2 ± 3.1
Medicine, LEDD	1031 ± 314
Medicine, agonists	14
UPDRS-III-OFF	27.9 ± 6.8

Handedness was assessed with the Edinburg Handedness Inventory. BIS-11 = Barratt impulsiveness scale; F = female; GDS-30 = geriatric depression scale, 30 items; LEDD = l-dopa–equivalent daily dose; MoCA = Montreal Cognitive Assessment; R = right; UDysRS = Unified Dyskinesia Rating Scale; UPDRS = Unified Parkinson’s Disease Rating Scale; ± standard deviation.

**Table 2:** Interventional TMS studies to improve LID in patients with Parkinson’s disease

First author, year Sample size Brain target	TMS protocol	Main findings	Clinical dyskinesia score	Score before TMS	Score after sham TMS	Score after real TMS	Pairwise testing (*P*-value)	ANOVA (*P*-value)	
Koch *et al.*, 2005 8 patients Target: SMA	Single rTMS session at 1 or 5 Hz for 15 min with continuous apomorphine infusion	1-Hz rTMS session reduced AIMS for 15 min after rTMS; 5-Hz rTMS session had no effect	AIMS	6.75 ± 2.0 6.00 ± 1.8	6.35 ± 1.9 6.35 ± 1.9	2.25 ± 1.8 7.38 ± 2.7	*P* < 0.01^a^	*P* < 0.00^b^ *P* < 0.12^b^	
Brusa *et al.*, 2006 10 patients Target: SMA	Single and multiple 1-Hz rTMS sessions for 15 min *after* levodopa intake	Single 1-Hz rTMS session reduced AIMS for 15 min after rTMS; Multiple 1-Hz rTMS sessions (1/day, 5 days) had same effect	AIMS	0.0 ± 0.0^c^ 0.0 ± 0.0^c^	6.62 ± 1.0 6.62 ± 1.0	1.37 ± 1.5 1.45 ± 1.4		*P* < 0.001^b^ *P* < 0.001^b^	
Lohse *et al.*, 2020 (data reported in this publication) 17 patients Target: PreSMA	Single 1-Hz rTMS session for 30 min *before* levodopa intake	Single-session 1 Hz reduced and delayed UDysRS	UDysRS Time to LID onset		12^d^ 24.5 min	9 36.5 min	*P* = 0.032^e^ *P* = 0.019^e^		
Flamez *et al.*, 2016 9 patients (single) 6 patients (multiple) Target: M1	Single bilateral 1-Hz rTMS session for 16 min *after* levodopa intake; Multiple bilateral 1-Hz rTMS sessions for 32 min during continued levodopa treatment	Single 1-Hz rTMS session did not produce an anti-dyskinetic effect Multiple sessions (2/day, 5 days) did not produce an anti-dyskinetic effect	AIMS AIMS UPDRSIV PDYS-26	0 ± 0 12 ± 5 4.8 ± 0.8 22 ± 11	8 ± 5 11 ± 5 4.7 ± 0.5 22 ± 12 (Day 5)	10 ± 5 8 ± 5 4.8 ± 0.8 22 ± 11 (Day 5)		*P* = 0.11^f^ *P* = 0.23^f^ *P* = 0.84^f^ *P* = 0.40^f^	
Wagle-Shukla *et al.*, 2007 6 patients Target: M1	Multiple 1-Hz rTMS sessions for 15 min *after* levodopa intake	Multiple sessions (1/day, 10 days in 2 weeks) reduced CAPSIT for 1 day after the last stimulation	CAPSIT-PD	5.6 ± 2.8	No sham	3.5 ± 1.6 (1 day after last rTMS)	*P* = 0.03^g^	*P* < 0.06^h^	
Filipović *et al.*, 2009 10 patients Target: M1	Multiple 1-Hz rTMS sessions for 32 min during continued levodopa treatment	Multiple sessions (1/day, 4 days) did not reduce CDRS on direct comparison	CDRS	22.1 ± 8.2	20.8 ± 6.7	20.4 ± 7.9	*P* = 0.22^i^	*P* < 0.043^j^	
Sayın *et al.*, 2014 17 patients Target: SMA	Multiple 1-Hz rTMS sessions for 30 min during continued levodopa treatment^k^	Multiple sessions (1/day, 10 days) reduced AIMS for 24 h after stimulation	AIMS	16.61 ± 6.23	9.56 ± 4.06^l^	13.72 ± 7.28	*P* = 0.024^m^		
Koch *et al.*, 2009 10 patients (single) 20 patients (multiple) Target: Cerebellum	Single cTBS sessions *after* levodopa intake; Multiple cTBS sessions *after* levodopa intake^k^	Single cTBS session reduced CAPSIT-PD; Multiple cTBS sessions (1/day, 10 days, in 2 weeks) reduced CAPSIT for up to 4 weeks after stimulations	CAPSIT-PD	∼ 6.5^n^	∼5.8^n^ ∼5.8^n^ (3 days after last rTMS)	∼3.8^n^ ∼3.8^n^ (3 days after last rTMS)	*P* = 0.011^o^ *P* = 0.008^p^	*P* < 0.007^o^ *P* < 0.0001^p^	
Cerasa *et al.*, 2015 11 patients Targets: right IFC and M1	Single cTBS session *after* levodopa intake	Single cTBS session of right IFG reduced AIMS for 30 min after stimulation; No effect of single cTBS session over M1	AIMS		6.33 ± 1.4 ∼5.0^q^	4.50 ± 1.3 ∼5.0^q^	*P* = 0.04^r^		
Ponzo *et al.*, 2016 10 patients Target: right IFC	Single cTBS session *after* levodopa intake	Single-session cTBS reduced AIMS relative to sham cTBS	AIMS	∼3.0^c^^,s^	∼7.0^s^	∼4.0^s^	*P* = 0.02^t^		

Data are presented as mean ± standard deviation. Studies are sorted by TMS protocol and thereafter by year. AIMS = Abnormal Involuntary Movement Scale; CAPSIT-PD = The Core Assessment Program for Surgical Interventional Therapies in Parkinson’s Disease; cTBS = continuous theta burst transcranial magnetic stimulation; IFC = inferior frontal cortex; M1 = primary motor cortex; PDYS-26 = 26-item Parkinson Disease Dyskinesia Scale; preSMA = pre-supplementary motor area; rTMS = repetitive transcranial magnetic stimulation; SMA = supplementary motor area; UDysRS = Unified Dyskinesia Rating Scale; UPDRS = Unified Parkinson’s Disease Rating Scale.

a Wilcoxon test of ‘score after real TMS’ versus ‘pre-TMS score’.

b Friedman ANOVA for repeated measures with time as the main effect for the entire 1-Hz TMS condition with multiple time points, not only the score reported in this table.

c The pre-score was measured immediately after levodopa intake in patients withdrawn from therapy since the night before.

d These are median UDysRS scores.

e One-tailed Wilcoxon test for ‘score after real TMS’ versus ‘score after sham TMS’.

f Two-way repeated measure ANOVA of interaction between stimulation (sham or real) as between-subject factor and time as within-subjects factor.

g Paired *t*-test. Scores are measured at peak-of-dose dyskinesia.

h Repeated measures ANOVA.

i One-tailed directional Wilcoxon-matched pairs test for ‘score after real TMS’ versus ‘score after sham TMS’ change from ‘pre-TMS score’.

j Friedman ANOVA for ‘pre-TMS score’ versus ‘score after real TMS’ versus ‘score after sham TMS’.

k This study used a parallel group design for the multi-session part of the study. All others used a one group cross-over design.

l The ‘pre-TMS score’ for the sham group was 8.31 ± 3.52.

m Wilcoxon test for ‘score pre-rTMS’ versus ‘score after real-rTMS’ both at 90 min after levodopa.

n Scores were estimated taken from Fig. 4.

o Respectively a Wilcoxon test and Friedman ANOVA for ‘score after real TMS’ versus ‘score after sham TMS’ both at 30 min after levodopa.

p Respectively a Wilcoxon test and Friedman ANOVA for the ‘score after real TMS’ versus the ‘pre-TMS score’ both at 30 min after levodopa.

q Scores were estimated taken from Fig. 4.

r Wilcoxon test for ‘score after real TMS’ versus ‘score after sham TMS’ both at 30 min after levodopa.

s Scores were estimated taken from Fig. 1D.

t Wilcoxon *t*-test for ‘score after real TMS’ versus ‘score after sham TMS’.

On the first experimental day, patients underwent a standardized neuropsychological and clinical assessment before they participated in the rTMS experiment. Motor symptoms of Parkinson’s disease were assessed with the Unified Parkinson’s Disease Rating Scale part III (UPDRS-III) and dyskinesia severity was assessed with the Unified Dyskinesia Rating Scale (UDysRS) in a typical ON-medication state. We also assessed handedness (Edinburgh handedness inventory) and impulsivity (Barratt impulsivity scale) and screened for cognitive impairment (Montreal Cognitive Assessment) and depression (geriatric depression scale, 30 items). Patients were also trained on the task to be performed on Days 3 and 4 during the main rTMS-fMRI experiment (see below). Patients underwent a structural MRI of the brain at 3 T for stereotactic targeting of preSMA on a second day, apart from a few patients who wished to have the clinical assessment and structural MRI scans to be performed on a single day.

### Go/no-go task

During task-fMRI, patients performed a visually cued go/no-go task (Fig. 1) (Herz *et al.*, 2014). The mean inter-trial interval was 3.5 s to give patients enough time to respond. At trial onset, a geometrical shape (black triangle, circle or square) was presented in the central visual field. A ‘left go’ and ‘right go’ stimulus instructed patients to click with their left or right index finger on an MRI-compatible mouse. The third stimulus was a ‘no-go’ cue, which prompted patients to withhold any motor response. The shape cue was replaced by a central fixation cross after 0.75 s, which stayed on the screen for 2.25–3.25 s before the next cue appeared. Visual stimuli were projected onto a screen from the back of the scanner and viewed by patients via a coil-mounted mirror. A single task-related fMRI run lasted 9 min and consisted of 150 pseudo-randomly ordered trials with an equal probability of ‘left go’, ‘right go’ and ‘no-go’ trials, corresponding to 289 echo planar imaging brain volumes.

**Figure 1:**
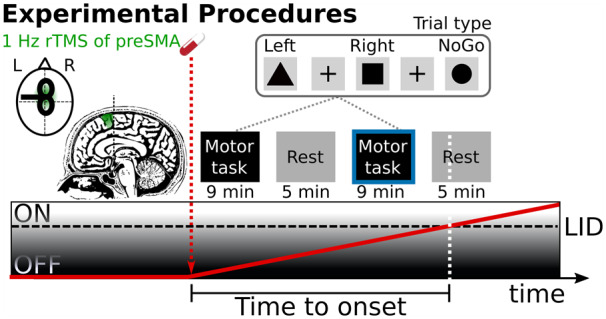
**Experimental procedures and timeline. Patients underwent robot-guided real or sham 1-Hz rTMS of preSMA in counterbalanced order on two separate days.** The preSMA was targeted with stereotactic neuronavigation using the peak activation from a previous fMRI study (Herz *et al.*, 2014) as target site [Montreal Neurological Institute (MNI) coordinates: *x*, *y*, *z *=* *4, 8, 58 mm]. Patients were OFF their usual dopamine replacement therapy. After 1-Hz rTMS of preSMA, patients received an oral solution of 200 mg levodopa and underwent task-based and resting-state fMRI runs in alternating order until patients developed peak-of-dose LID. During task-based fMRI, patients performed a stimulus-response mapping task. The fMRI analysis focussed on the last completed run before the emergence of LID (blue border).

### Study design

This interventional study had a within-subject, cross-over design. Patients were enrolled by Al.L. and treated with real- or sham-rTMS on 2 days separated by at least 2 weeks in a counterbalanced order. The order of the treatments for the individual patient was assigned serially by Al.L. based on a list of randomly ordered ones and twos generated by Al.L. in MATLAB ahead of the data collection. The time line of a single experimental session is illustrated in Fig. 1. Apart from an additional rTMS session between the OFF- and ON-medication scans, the timeline was identical to that of Herz *et al.* (2014) to facilitate a direct comparison.

The study was conducted at the Danish Research Centre for Magnetic Resonance, Hvidovre Hospital, Denmark from August 2017 until September 2018. Patients were transferred to the scanner room in an MR compatible wheelchair immediately after preSMA stimulation. Patients received an oral solution of 200 mg levodopa in combination with 50 mg benserazide (Madopar Quick^®^, La Roche). We considered to adjust the levodopa dose to the individual l-dopa–equivalent daily dose but eventually decided to give a fixed dose to secure comparability with our previous pharmacological fMRI study (Herz *et al.*, 2014). Patients were positioned in the MRI scanner and task-related brain activity was mapped with fMRI until the emergence of dyskinesia. Task-based fMRI runs were interleaved with 5 min of resting-state fMRI. This cycle of task- and resting-state-fMRI was repeated until choreiform movements became visible to the investigator positioned inside the scanner room (Al.L.). If patients did not develop choreiform dyskinesia within four cycles of task- and resting-state-fMRI the session was stopped.

Patients were assessed clinically for symptoms of Parkinson’s disease (UPDRS-III) and dyskinesia (UDysRS) prior to the stimulation and immediately after the scanning session. The assessment was video recorded for subsequent blinded scoring of the UPDRS-III (except rigidity) and the objective part of the UDysRS. Blinded scoring was performed by a movement disorder specialist (An.L.).

### Robot-assisted transcranial magnetic stimulation

Real and sham 1-Hz rTMS was applied through a figure-8 coil **(**MCF-B65; Ø 2 × 75 mm) using a MagPro X100 with MagOption stimulator (Magventure, Farum, Denmark). A total number of 1800 biphasic TMS pulses were continuously applied to preSMA at a repetition rate of 1 Hz for 30 min. For real-rTMS, stimulation intensity was set to 100% of the resting motor threshold of the left first dorsal interosseous muscle, while stimulation intensity was set to 30% of individual resting motor threshold in the sham-rTMS session. Individual resting motor threshold was determined using a maximum-likelihood strategy implemented in the TMS Motor Threshold Assessment Tool (MTAT 2.0; http://www.clinicalresearcher.org/software.htm) with the centre of the coil placed over the cortical motor hot spot of the left first dorsal interosseous muscle. Motor evoked potentials were judged to be present when peak-to-peak motor evoked potential amplitude exceeded 50 µm.

PreSMA was targeted using a frameless stereotactic neuronavigation system (LOCALITE, Sankt Augustin, Germany) based on the patient’s individual T_1_-weighted MRI brain scans. After normalizing the patient’s T_1_-weighted brain scan into standard MNI space, the coil was positioned over the preSMA with the coil centre projecting on the MNI-coordinates *x*, *y*, *z *=* *4, 8, 58, which corresponded to the activation peak revealed by our previous study (Herz *et al.*, 2014). The coil was positioned in a way that the second (most effective) phase of the biphasic TMS pulse always had a left-to-right current direction in the preSMA. During the rTMS intervention, a constant coil position was automatically maintained by a robot (Axilium Robotics, Strasbourg, France).

### Structural and functional MRI acquisition

Structural and functional MRI scans were acquired on a 3-T Verio scanner with a 32-channel head coil (Siemens, Erlangen, Germany). The MRI acquisition protocols were identical to those of Herz *et al.* (2014). A structural T_1_-weighted brain scan used a magnetization prepared rapid gradient echo sequence (field-of-view: 230 mm, slice thickness: 0.9 mm, repetition time: 1900 ms, echo time: 2.32 ms and flip angle: 9°). Blood oxygen level dependent fMRI employed a T_2_*-weighted echo planar imaging sequence (field-of-view: 192 mm, slice thickness: 3.5 mm, slice spacing: 0.2 mm, repetition time: 1850 ms, echo time: 26 ms and flip angle: 75°).

#### Analysis of clinical outcome measures and task performance

Statistical analyses of clinical outcome measures and task performance were performed using R software (R Foundation for Statistical Computing, Vienna, Austria). To test whether real-rTMS of preSMA had an acute beneficial effect on LID, we compared the time from levodopa intake to the onset of dyskinesia as well as the severity of dyskinesia after real- and sham-rTMS. Since the data violated the assumptions for parametric testing, we used one-tailed Wilcoxon signed-rank tests to test the pre-registered hypotheses that real-rTMS prolongs the time from levodopa intake to the onset of dyskinesia and decreases dyskinesia severity compared with sham-rTMS. The median and interquartile range are reported to describe the group data. We also computed exploratory correlational analyses to test whether individual improvements in dyskinesia, reflected by a later onset or reduced severity, scaled with the induced electrical field strength or with a change in task-related activity in the targeted preSMA. Results of the correlational analyses are reported as Pearson correlation coefficients.

We also compared task performance during fMRI, focussing on the response times during go trials and errors of commission in no-go trials with paired *t*-tests. Response times were log-transformed to meet assumption of normal distribution. Significance threshold was set at *P* < 0.05. Behavioural group data are reported as mean ± SD.

### Functional MRI data analysis

We analysed the last completed fMRI run before peak-of-dose dyskinesia emerged. Imaging analysis was performed in MATLAB (Release 2018a, The MathWorks, Inc., Natick, MA, USA) using the SPM12 toolbox (revision number 6906, The Wellcome Centre for Human Neuroimaging, UCL, London, UK). The three first volumes of each fMRI run were discarded to allow for T1 equilibrium effect. The echo planar imaging volumes from the real-rTMS and sham-rTMS run were realigned to the mean echo planar imaging images of the respective run, co-registered to the individual structural T_1_-weighted scan, spatially smoothed at full-width half-maximum of 8 mm and high-pass filtered (1/128 Hz). Brain volumes were excluded when the relative movement with respect to the previous volume exceeded 1 mm or the motion threshold, the absolute change in mean signal from volume to volume exceeded the value of 1 (Power *et al.*, 2012).

Three patients developed dyskinesias already during the first run after levodopa intake: one patient after real-rTMS, one patient after sham-rTMS and one patient after both real- and sham-rTMS. For these patients, only the non-dyskinetic part of the fMRI runs was used for further analysis. For the remainder of the patients, the last complete task-fMRI run *before* the run in which the patients developed dyskinesia was used for the analysis.

A general linear model was specified to perform a voxel-wise analysis for the sham- and the real-rTMS runs. Five task events were modelled as separate regressors: left go, right go, no-go, no-go error and late no-go error. Errors were considered late when response times exceeded the stimulus presentation time (750 ms). A first-order linear time modulation was modelled for the first three regressors of interest in order to model gradual changes in activity over time induced by levodopa. Twenty-four regressors of no interest were specified to account for residual movement artefacts, as well as regressors modelling pulsation and respiration (Friston *et al.*, 1996). For each participant, a *t*-test was run to test for differences in linear changes over time in neural activity during no-go responses between the sham- and the real-rTMS session.

The individual t-contrast maps were entered into a random-effects second-level general linear model analysis. We also tested whether the change in task-related brain activity induced by real-rTMS scaled linearly with the acute clinical effect of real-rTMS on dyskinesia or the electrical field strength induced by real-rTMS in the targeted cortex (i.e. the stimulation dose). We applied a cluster-forming threshold of *P *<* *0.001 (uncorrected) and applied the family-wise error method to correct for multiple comparisons at the cluster level (*P *<* *0.05). We pre-specified four regions of interest (ROIs), including the preSMA, the pars opercularis of the inferior frontal gyrus (IFG), putamen and subthalamic nucleus (STN). All ROIs were merged into a combined bi-hemispheric mask and family-wise error correction was restricted to the voxels within the mask covering all four ROIs. The preSMA region was defined based on the on description in Johansen-Berg *et al.* (2004), comprising the frontal medial wall of the hemispheric cortex with the posterior border corresponding to the vertical anterior commissure line and the anterior border corresponding to the vertical plane defined by the *y* coordinate 30. The ventral border was marked by the cingulate sulcus. The IFG and putamen ROIs were defined using the automatically labelled anatomical masks from the WFU pick Atlas (Maldjian *et al.*, 2003). The STN ROI was defined using the probabilistic mask based on 7-T MRI (Keuken *et al.*, 2014).

### Electrical field simulation

The structural MRI images were used to model the distribution of the electric field induced by real-rTMS targeting preSMA (SimNIBS software, v. 2.1.1, http://simnibs.org). Estimation of the TMS-induced electric field was based on the finite element method, using individualized tetrahedral head meshes generated from the individual structural MR image of each participant (Windhoff *et al.*, 2013; Thielscher et al., 2015). Head reconstruction was performed using the incorporated headreco tool based on SPM12 and CAT12 toolboxes with the *–d no-conform* option enabled (Nielsen *et al.*, 2018).

We used the SimNIBS graphical user interface to set up the electrical field simulations placing the TMS coil at the position obtained from the Localite neuronavigation system and the d*I*/d*t* (current change ratio) for each subject. The coil model was chosen to match the Magventure MCF-B65 stimulation coil. The simulated electrical field maps were then resampled onto the FreeSurfer FSAverage brain template (https://surfer.nmr.mgh.harvard.edu). To estimate the efficacy of electrical stimulation in the target region, we calculated the mean electrical field strength from a sphere with a 20-mm radius around the stimulation target, i.e. MNI-152 coordinates *x*, *y*, *z* = 4, 8, 58 (mm). We performed linear regression analysis to test whether the TMS-induced electrical field strength in the targeted preSMA predicted the interventional effect of real-rTMS as reflected by the time from levodopa intake to dyskinesia onset or the severity of LID.

### Data availability

Data from this study are available on request from the author but require a data sharing agreement in accordance with Danish and European data protection law. The data are not publicly available because of local ethical restrictions and protection of privacy of study participants.

## Results

All 17 patients included in the final analysis experienced mild to moderate dyskinesia following levodopa intake. One patient did not develop dyskinesia inside the scanner during the MRI sessions, but mild dyskinesia emerged outside the MR scanner shortly after the end of both MRI measurements. A second patient only developed dyskinesia outside the scanner after the real-rTMS session, but inside the scanner during the sham-rTMS session. A third patient showed the opposite pattern. The time to onset of dyskinesia was not correlated with their severity in neither the sham-rTMS condition (*ρ* = 0.28, *P *=* *0.3) nor the real-rTMS condition (*ρ* = 0.2, *P *=* *0.48). Hence, a shorter time to onset of dyskinesia was not associated with stronger peak-of-dose dyskinesia.

### Clinical effects of preSMA stimulation

**Figure 2:**
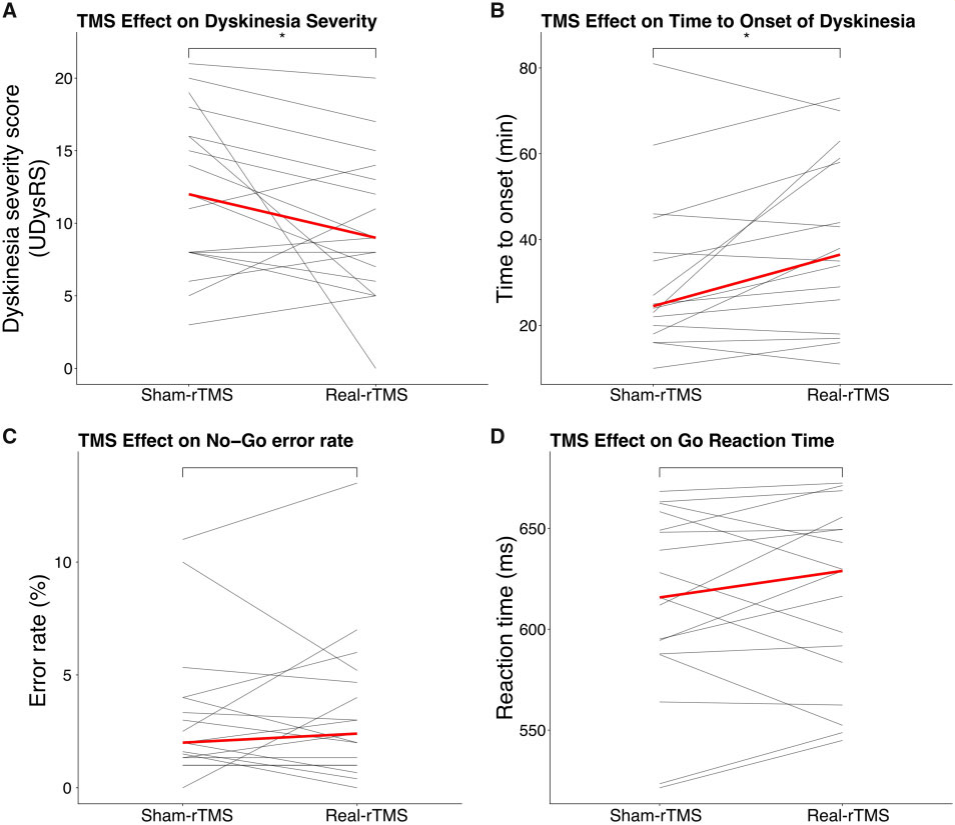
**Inhibitory 1-Hz rTMS of preSMA improves clinical outcome variables in a dose-dependent manner.** (**A**) TMS effect on dyskinesia severity (UDysRS). (**B**) TMS effect on the time from levodopa intake to the onset of dyskinesia. (**C**) Error rate (no-go commission errors). (**D**) Reaction times for go trials. *P <* *0.05 (one-sided Wilcoxon signed-rank test). Red line represents the median.

**Figure 3:**
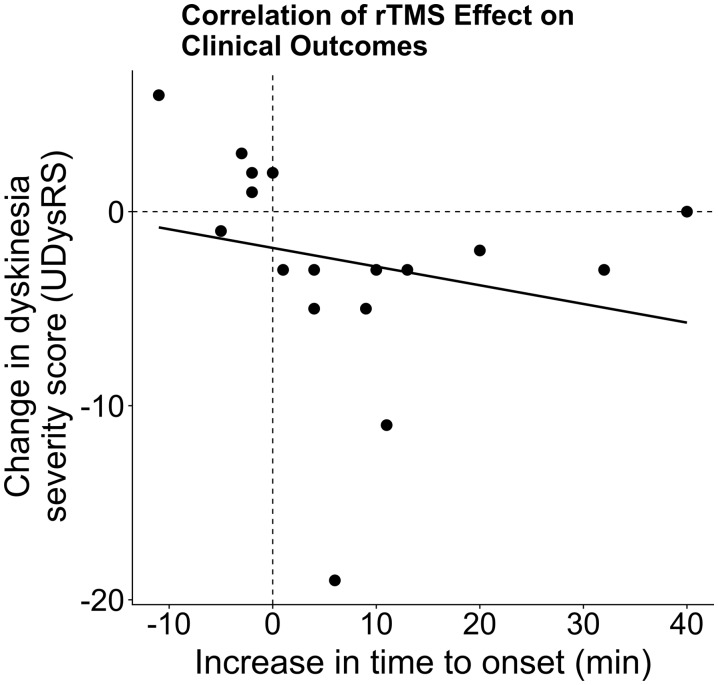
**Correlation of changes in clinical outcome measures after real-rTMS.** Across the group, increases in time to onset were significantly correlated with decreases in dyskinesia severity (*r *=* *0.72, *P *=* *0.002). UDysRS, Unified Dyskinesia Rating Scale.

A single session of robot-assisted real-rTMS applied over preSMA had an acute beneficial effect on peak-of-dose dyskinesia compared with sham-rTMS (Fig. 2). Dyskinesia severity decreased by 25% at the group level, corresponding to a reduction of median UDysRS score by 3.0 points *(W* = 32; *P*(one-tailed) = 0.032; Fig. 2A). Real-rTMS also prolonged the time from intake of levodopa to onset of dyskinesia by 20% *(W* = 108.5; *P*(one-tailed) = 0.019; Fig. 2B). The median time from levodopa intake to dyskinesia onset increased by 5 min. The real-rTMS-induced reduction in dyskinesia severity showed a linear relationship with the real-rTMS-induced prolongation of the time to dyskinesia onset (*r *=* *0.72, *P *=* *0.002; Fig. 3). The beneficial effect of real-rTMS on dyskinesias was not paralleled by an attenuation of the therapeutic anti-parkinsonian effects of levodopa. The levodopa-induced reduction in motor UPDRS-III scores was not significantly different in the two experimental sessions with a mean reduction in UPDRS-III score of 7.5 ± 5.3 after real-rTMS and 8.5 ± 4.5 after sham-rTMS (*W* = 60.5; *P *=* *0.64).

Measurement of task performance during task-related fMRI revealed a low rate of commission errors in no-go trials. Overall, mean error rate was 3.2% and did not differ between the real and sham-rTMS **(***t*(16) = 0.28; *P *=* *0.79**)**, showing that real-rTMS did not affect the ability to withhold a motor response. Overall, rTMS did not affect go reaction times (mean difference 3.42 ms, *t*(16) = 0.47, *P *=* *0.65).

### Impact of stimulation dose on the anti-dyskinetic effect of preSMA stimulation

**Figure 4:**
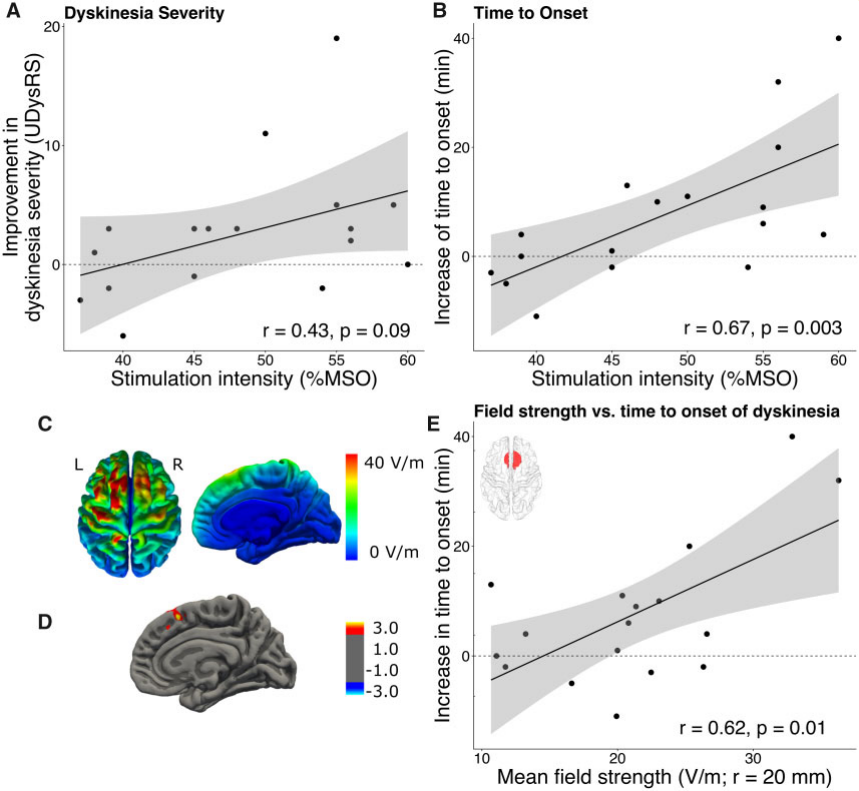
**Dose-dependent effects of rTMS on clinical outcome measures.** (**A**) Association between stimulation intensity and improvement in dyskinesia severity. (**B**) Association between stimulation intensity and prolongation of time between levodopa intake and the onset of dyskinesia. (**C**) Group mean rTMS-induced electrical field. (**D**) The rTMS-induced delay in dyskinesia onset correlated positively with the induced electrical field strength in preSMA. (**E**) Correlation between rTMS-induced delay in dyskinesia onset and mean induced electrical field strength in a sphere (*r* = 20 mm) centred at the stimulation target (MNI *x*, *y*, *z *=* *4, 8, 58). Shaded areas around linear fits represent the 95% confidence interval.

While the beneficial effects of real-rTMS of preSMA were statistically significant, the individual responses to the stimulation varied considerably between patients. This between-patient variability in responsiveness to real-rTMS scaled positively with the stimulation intensity, expressed as percentage of maximum stimulator output. We found a significant linear correlation between stimulation intensity and individual shortening of the time to onset of dyskinesia after levodopa intake (*r *=* *0.67, *P *=* *0.003; Fig. 4B). A similar trend was visible between stimulation intensity and individual reduction in dyskinesia severity, but it did not reach statistical significance (*r *=* *0.43, *P = *0.09; Fig. 4A).

We explored the relationship between the ‘dose’ of real-rTMS of preSMA and its acute anti-dyskinetic effect in more detail performing realistic simulations of the induced electrical fields based on the individual structural MRIs (Fig 4, lower panels). While real-rTMS induced the strongest electrical fields superficially in the crowns of frontal gyrus close to the midline, the induced field strength in these areas did not scale with the anti-dyskinetic effect of real-rTMS (Fig. 4C). In contrast, the induced electrical field strength in a well-defined spot of the preSMA and the pericentral cortex corresponding to the hand knobs showed a positive linear relationship with the delay of dyskinesia onset after real-rTMS (Fig. 4C). The linear relationship between the induced electrical field and time to dyskinesia onset peaked at *x*, *y*, *z* coordinates of 8, 19, 52 mm in preSMA. A positive linear effect of the regional dose induced in the preSMA on the time to dyskinesia onset was confirmed by a ROI analysis based on the mean induced electrical field strength in the preSMA target region (Fig. 4D). No such association was found between the induced regional electrical field strength and the anti-dyskinetic effect of real-rTMS on dyskinesia severity.

### Stimulation-induced change in task-related no-go activity

**Figure 5:**
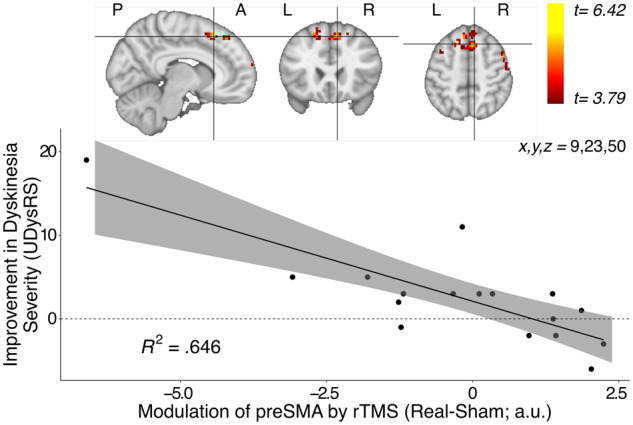
**Linear increase in neural activity during no-go trials covarying with rTMS-induced improvement in dyskinesia severity.** (**A**) During no-go trials, preSMA activity parametrically modulated by time covaried with rTMS-induced improvement in dyskinesia. The largest effect was in the anterior part of the right preSMA (peak *Z*-score = 4.26, *x*, *y*, *z *=* *9, 23, 50, *P *=* *0.002, cluster extent = 105 voxels) (**B**) The patients who showed the largest improvement in dyskinesia severity scores following real-rTMS had a lower increase of their preSMA activity for no-go trials over time compared with sham-rTMS.

We used task-based fMRI to test whether the anti-dyskinetic effect of real-rTMS was paralleled by a functional change in the targeted preSMA, indicating effective target engagement. In our previous task-based fMRI study, we had found an association between the emergence of levodopa-induced dyskinesia and an excessive increase in no-go activity in preSMA after levodopa intake (Herz *et al.*, 2014). Therefore, we focussed our analysis on the linear change of no-go activity over time in the preSMA in the fMRI run prior to dyskinesia onset. The relative change of preSMA no-go activity after real-rTMS scaled with the individual improvement in dyskinesia severity. The larger the improvement in dyskinesia severity scores after real-rTMS, the more no-go activity in preSMA was reduced by real relative to sham-rTMS (Fig. 5). The linear relationship between the anti-dyskinetic effect and the suppressive effect on no-go activity peaked in the anterior part of the right preSMA (peak z-score = 4.26, *x*, *y*, *z *=* *9, 23, 50, *P *=* *0.002, cluster extent = 105 voxels). While we have no reason to assume that the participant showing the strongest negative TMS-induced modulation of preSMA activity and the strongest improvement in dyskinesia severity does not truthfully represent the relationship between these two variables, we also tested for the relationship without this participant. This rendered the effect non-significant at *P* < 0.001 (uncorrected). However, a very similar pattern of activity modulation was still visible at a lower threshold of *P* < 0.05 (uncorrected), suggesting that the association was not artificially induced by only this participant, albeit with the peak *Z*-score now being located in left preSMA (*Z*-score = 2.90, *x*, *y*, *z* = −15, 26, 53, Supplementary Fig. 1).

A similar relationship was not found in the remaining three pre-specified ROIs including IFG, putamen and STN. No association was found between the real-rTMS-induced alteration of no-go activity in preSMA and the time from levodopa intake to dyskinesia onset.

## Discussion

We found that a single session of robot-assisted inhibitory 1-Hz rTMS (real-rTMS) targeting the preSMA acutely improved peak-of-dose dyskinesia in Parkinson’s disease. Real-rTMS of preSMA delayed and attenuated the dyskinesia-provoking effect of a single dose of levodopa compared with sham-rTMS. The more dyskinesia onset was delayed, the more severity of dyskinesia was suppressed. In the following, we first point out how the results may help to explain how dyskinesias originate at the circuit level after levodopa intake. We then discuss our findings considering the segregation of SMA into rostral preSMA and caudal SMA proper. We conclude with taking a therapeutic perspective and relate our findings to previous work that have used transcranial brain stimulation as interventional tool to improve LID.

### A ‘dual-circuit’ model underpinning peak-of-dose dyskinesia

Multiple lines of research point to a critical role of the posterior putamen and a resulting circuit dysfunction of the motor cortico-basal ganglia thalamocortical circuit for the emergence of LID in Parkinson’s disease. The classical notion is that progressive nigrostriatal neurodegeneration and prolonged dopamine replacement therapy cause maladaptive, non-homeostatic synaptic plasticity in spiny projection neurons of the motor striatum (Cenci and Konradi, 2010). This gives rise to a functional imbalance between the efferent direct and indirect pathways in the motor cortico-basal ganglia circuits (Barroso-Chinea and Bezard, 2010; Picconi *et al.*, 2018) and causes disruptions of neuronal ensemble activity (Zhai *et al.*, 2019) depending on motor activity. A multi-tracer positron emission tomography study found that levodopa produced a stronger dissociation of metabolic and neurovascular effects (which are normally coupled) in posterior putamen in Parkinson’s disease patients with LID compared to patients without LID (Jourdain *et al.*, 2016). Altered activity in the motor putamen and its efferent striatal pathways leads to reduced and aberrant activity in the STN, internal globus pallidus and precentral motor cortical areas, including the primary motor cortex and caudal SMA, in the dyskinetic ON-medication state (Rascol, 1998; Obeso *et al.*, 2000).

However, several pieces of evidence suggest that the pathophysiology of peak-of-dose dyskinesia goes beyond the motor basal ganglia loop. Mitchell *et al.* (1989, 1992) applied the 2-deoxyglucose tracer method to map regional metabolic changes induced by dopamine depletion and subsequent dopaminergic treatments in monkeys. The most affected structure was the STN, which displayed increased levels of 2-deoxyglucose uptake. Increased metabolism was most prominent in the ventromedial non-motor portion of the STN, which belongs to the associative and limbic basal ganglia loop. Importantly, the increase in subthalamic 2-deoxyglucose uptake was greater in animals showing peak-dose dyskinesia than in those without dyskinesia. These findings were later confirmed and extended by Guigoni *et al.* (2005) who postulated a link between the manifestation of dyskinesia and a pathological processing of limbic and cognitive information. In accordance with these findings, above-mentioned multi-tracer positron emission tomography study showed that the dissociation of vasomotor and metabolic drug responses to levodopa was not restricted to the posterior ‘motor’ putamen but extended rostrally into the ‘associative’ putamen in Parkinson’s disease patients with LID (Jourdain *et al.*, 2016).

Additional evidence for a relevant involvement of non-motor basal ganglia circuits in the pathophysiology of peak-of-dose dyskinesia points especially towards the inhibitory control network with its three core regions, the right IFG, preSMA and STN (Aron and Poldrack, 2006; Aron *et al.*, 2007; Cavanagh *et al.*, 2014). The preSMA and IFG are thought to generate a global ‘braking’ signal in the basal ganglia circuits that can pause or slow down motor execution in the context of surprise, conflict or errors (Aron *et al.*, 2007). This global no-go signal is fed into the basal ganglia via the hyperdirect pathway to the STN (Frank, 2006; Aron *et al.*, 2016). Accordingly, healthy individuals who show stronger task-related activity in the anterior preSMA have been found to be better at response stopping (Ray Li *et al.*, 2008). It also accounts for a gradual increase in activity in the preSMA when healthy individuals take increasingly risky actions during a sequential gambling task (Meder *et al.*, 2016).

Evidence for preSMA involvement in the aetiology of LID comes from our pharmacological fMRI study (Herz *et al.*, 2014, 2015, 2016). Parkinson’s disease patients with LID showed an excessive activity increase in preSMA and middle putamen during no-go trials, but not in motor executive areas, such as primary motor cortex or SMA. Effective connectivity analyses revealed that levodopa intake modulated the reciprocal connections from the putamen to primary motor cortex and to preSMA (Herz *et al.*, 2015). The current results extend the previous findings by showing that the ability to reduce no-go activity in preSMA predicted how much a patient would show a reduction in dyskinesia severity. The overactivation of preSMA in Parkinson’s disease patients with LID seen in previous studies (Herz *et al.*, 2014, 2015, 2016) could either be detrimental, contributing to LID, or beneficial, suppressing the emergence of dyskinesia. Although mutually exclusive, both hypotheses are conceivable, because the preSMA has been implicated in both, the suppression and initiation of movements (Fried *et al.*, 1991; Ikeda *et al.*, 1999). Our results strongly support the notion that excessive activity found in preSMA is a maladaptive response to levodopa that promotes the emergence of dyskinesia.

The other main cortical node of the inhibitory control network, the right IFG, has also been implicated in the emergence of LID in Parkinson’s disease. Two studies reported significant improvement in dyskinesia severity after inhibitory continuous theta burst stimulation of right IFG (Cerasa *et al.*, 2015; Ponzo *et al.*, 2016). There are dense cortico-cortical connections between preSMA and right IFG (Bozkurt *et al.*, 2017), and both cortical areas are connected to the cerebellum via the STN and pontine nuclei, which feeds back to the basal ganglia via the dentato-thalamo-striatal pathway (Bostan *et al.*, 2013). Hence, interventional rTMS of preSMA or IFG may target different entry nodes to the same dysfunctional inhibitory network (Aron *et al.*, 2007). Given the anatomical connectivity, our results suggest that each node of the inhibitory network may qualify as target for rTMS-based treatment of LID in patients with Parkinson’s disease. Further comparative studies are needed to establish, which target location or combination of target locations is most efficient to ameliorate LID.

We argue that in patients with LID, the associative (cognitive) basal ganglia circuit, which provides input to preSMA and IFG, becomes progressively oversensitive to levodopa, which adversely affects the patient’s ability to employ inhibitory cognitive control. The co-existing excessive responsiveness of the motor and associative (cognitive) basal ganglia circuits to levodopa affects the processing of cognitive and motor information and jointly reduces the threshold for levodopa to induce dyskinesias.

### Targeting the preSMA versus targeting the SMA proper

The SMA has been previously targeted with rTMS in Parkinson’s disease patients with LID, showing beneficial effects after a single session of 1-Hz rTMS (Koch *et al.*, 2005) and at the end of a treatment course with single sessions repeated over several days (Brusa *et al.*, 2006; Sayın *et al.*, 2014). Since previous studies did not differentiate between the rostral preSMA and caudal SMA proper, it remains unclear whether the beneficial effect on LID was caused by neuromodulatory effects of rTMS on preSMA or SMA proper or a combined effect on both areas.

The SMA proper and preSMA differ substantially in terms of their cytoarchitecture, structural and functional connectivity as well as activity (Matsuzaka *et al.*, 1992; Ikeda *et al.*, 1999; Johansen-Berg *et al.*, 2004; Nachev *et al.*, 2008; Kim *et al.*, 2010; Ruan *et al.*, 2018). In non-human primates, SMA proper and preSMA are only poorly connected by cortico-cortical fibres (Luppino *et al.*, 1993). The SMA proper is considered an executive motor area and as such part of the motor basal ganglia circuit, whereas the preSMA is thought to be a prefrontal area involved in abstract strategic goal implementation and action control (Ruan *et al.*, 2018).

Informed by our previous pharmacological fMRI study on LID (Herz *et al.*, 2014), we primarily targeted rostral preSMA. We attribute the beneficial effects of 1-Hz rTMS to a neuromodulatory effect specifically on preSMA based on three considerations. First, personalized coil placement guided by frameless stereotaxis secured in each patient that real-rTMS targeted the region in preSMA that previously had shown excessive task-related no-go activity in patients with LID (Herz *et al.*, 2014). Second, the magnitude of the induced electrical field in the preSMA target region, but not in caudal SMA proper or other mesial premotor regions, scaled positively with the rTMS-induced delay in dyskinesia onset. Third, the ability of rTMS to reduce excessive no-go activity in preSMA predicted its anti-dyskinetic effect at the individual level. No such relationship between functional ‘target engagement’ and LID outcome emerged in the caudal SMA or other motor areas.

Another way to probe target engagement would be to assess how 1-Hz rTMS changed the functional connectivity between the targeted region and connected brain regions, for instance the functional interaction between the targeted preSMA and the right IFG or STN. A large body of previous neuroimaging work has linked changes in functional cortico-basal ganglia connectivity to the expression of clinical symptoms such as LID or tremor in Parkinson’s disease (Cerasa *et al.*, 2015; Herz *et al.*, 2015, 2016; Dirkx *et al.*, 2017, 2020). Therefore, the use of neuroimaging to capture stimulation-induced changes in connectivity in the targeted brain circuit is warranted in future studies, preferably in conjunction with a local read-out of regional activity changes in the target area.

In conclusion, the beneficial effect of inhibitory 1-Hz rTMS of preSMA on peak-of-dose dyskinesia in LID patients supports a ‘double-hit’ or ‘dual-circuit’ hypothesis of the emergence of LIDs, pointing to a causal contribution of the mesial cortico-basal ganglia-thalamo-cortical associative circuits involving the preSMA in addition to the well-known dysfunction of the motor cortico-basal ganglia-thalamocortical circuits (Barroso-Chinea and Bezard, 2010; Cenci and Konradi, 2010; Picconi *et al.*, 2018). The dual-circuit hypothesis also predicts that a combined stimulation of preSMA and SMA proper may augment the therapeutic potential of interventional rTMS, because such an intervention would engage both dysfunctional mesial basal ganglia circuits.

### Transcranial stimulation to treat peak-of-dose dyskinesia

The neuroimaging results provide some indications how to optimize rTMS of preSMA in future therapeutic studies. In those patients, in whom real-rTMS had the strongest anti-dyskinetic effect, real-rTMS also induced the strongest electrical fields in the preSMA. This raises the possibility that only some patients may have received real-rTMS at a sufficiently high intensity. In this study, we adjusted stimulus intensity according to the individual corticomotor threshold of intrinsic hand muscles to better relate TMS dose to existing safety guidelines (Rossi *et al.*, 2009). To avoid underdosing, future studies may adjust stimulus intensities to the motor threshold of the motor leg area, which has a similar distance to the scalp surface as preSMA instead of the motor hand area. However, it is preferable to compute realistic electrical field simulations using the patient’s individual structural MR image. Based on these simulations, one can derive the individual stimulus intensity that is needed to produce a sufficiently strong electrical field in the preSMA (e.g. >25 V/m). Such a dosing procedure would ensure that 1-Hz rTMS induces a comparable stimulation in the target region and hereby increase the consistency of its anti-dyskinetic effects.

Beyond appropriate dosing in terms of stimulus intensity, task-based fMRI may reveal how efficiently the rTMS intervention functionally engaged the targeted brain region. In our study, pharmacodynamic fMRI measurements revealed that no-go activity in the stimulated preSMA predicted the anti-dyskinetic response to 1-Hz rTMS. In future therapeutic trials, the rTMS-induced modulation of no-go activity in preSMA may serve as functional read-out to drive an iterative process to adapt rTMS based on functional target engagement that has been achieved in previous rTMS sessions.

While the present results are encouraging, more research is needed to transform interventional rTMS into a clinically applicable treatment for LID. One lesson that has been learned from the use of rTMS as a therapeutic tool in patients with therapy-resistant depression is that repeated stimulation sessions are needed to induce clinically relevant treatment effects (Lefaucheur *et al.*, 2014). To qualify as a treatment, it needs to be shown that repeated sessions of rTMS to preSMA can induce a more sustained anti-kinetic response.

A central question that remains to be clarified is which brain region should be targeted with rTMS. Expanding the existing literature on the beneficial effects of focal rTMS on LID in Parkinson’s disease, this study identifies and validates the preSMA as promising target for stimulation therapies. Table 2 summarizes the key features of 10 published rTMS studies. All studies targeted a single brain region and applied inhibitory stimulation protocols (Table 2). Besides the targeting the SMA to improve LID, other studies have used transcranial brain stimulation of primary motor cortex (Wagle-Shukla *et al.*, 2007; Filipović *et al.*, 2009,  2013; Cerasa *et al.*, 2015), right IFG (Cerasa *et al.*, 2015) and the cerebellum (Koch *et al.*, 2009; Ferrucci *et al*., 2016). These areas are viable targets for two reasons. First, these targets are located sufficiently close to the brain surface so that they can be targeted with transcranial stimulation. Second, these regions are thought to become hyperactive and dysfunctional when patients take anti-parkinsonian dopaminergic medication where inhibitory rTMS thus may be beneficial (Meder *et al.*, 2019).

Apart from target site, existing studies also differ in many other aspects, including the stimulation protocol, number of sessions, medication state at time of stimulation and outcome measures. The considerable heterogeneity renders a direct comparison difficult and precludes conclusions, which of the targeted brain regions might be most promising as target for anti-dyskinetic stimulation therapies (Table 2). Therefore, future transcranial stimulation studies need to directly compare the anti-dyskinetic effects that can be achieved with brain stimulation of a single cortical target or with multi-site targeting.

## Conclusion

Supporting a dual-circuit model of LID in Parkinson’s disease, our results corroborate a causal role of preSMA in the pathophysiology of levodopa-induced peak-of-dose dyskinesia and show an anti-dyskinetic therapeutic potential of 1-Hz rTMS targeting preSMA. The individual anti-dyskinetic effect of 1-Hz rTMS scaled positively with rTMS-induced electrical field strength and with rTMS-induced attenuation of no-go activity in preSMA. These findings show that the combination of therapeutic rTMS and brain imaging can advance the mechanistic understanding of the observed therapeutic effects and help to generate testable hypotheses regarding the future optimization of rTMS as therapeutic intervention.

## Supplementary material

Supplementary material is available at *Brain Communications* online.

## Supplementary Material

fcaa147_Supplementary_Data

## Abbreviations

fMRI =: functional magnetic resonance imaging;

IFG =: inferior frontal gyrus;

LID =: levodopa-induced dyskinesia;

preSMA =: pre-supplementary motor area;

ROI =: region of interest;

rTMS =: repetitive transcranial magnetic stimulation;

STN =: subthalamic nucleus
