# Supplementary material for: Low-frequency transcranial stimulation of pre-supplementary motor area alleviates levodopa-induced dyskinesia in Parkinson’s disease: a randomized cross-over trial
Source: Brain Commun. 2020 Sep 18;2(2):fcaa147. doi: 10.1093/braincomms/fcaa147 (PMC7667528; doi:10.1093/braincomms/fcaa147)
Supplement: fcaa147_Supplementary_Data [file fcaa147_supplementary_data.zip › Supplementary Figure 1.pdf]

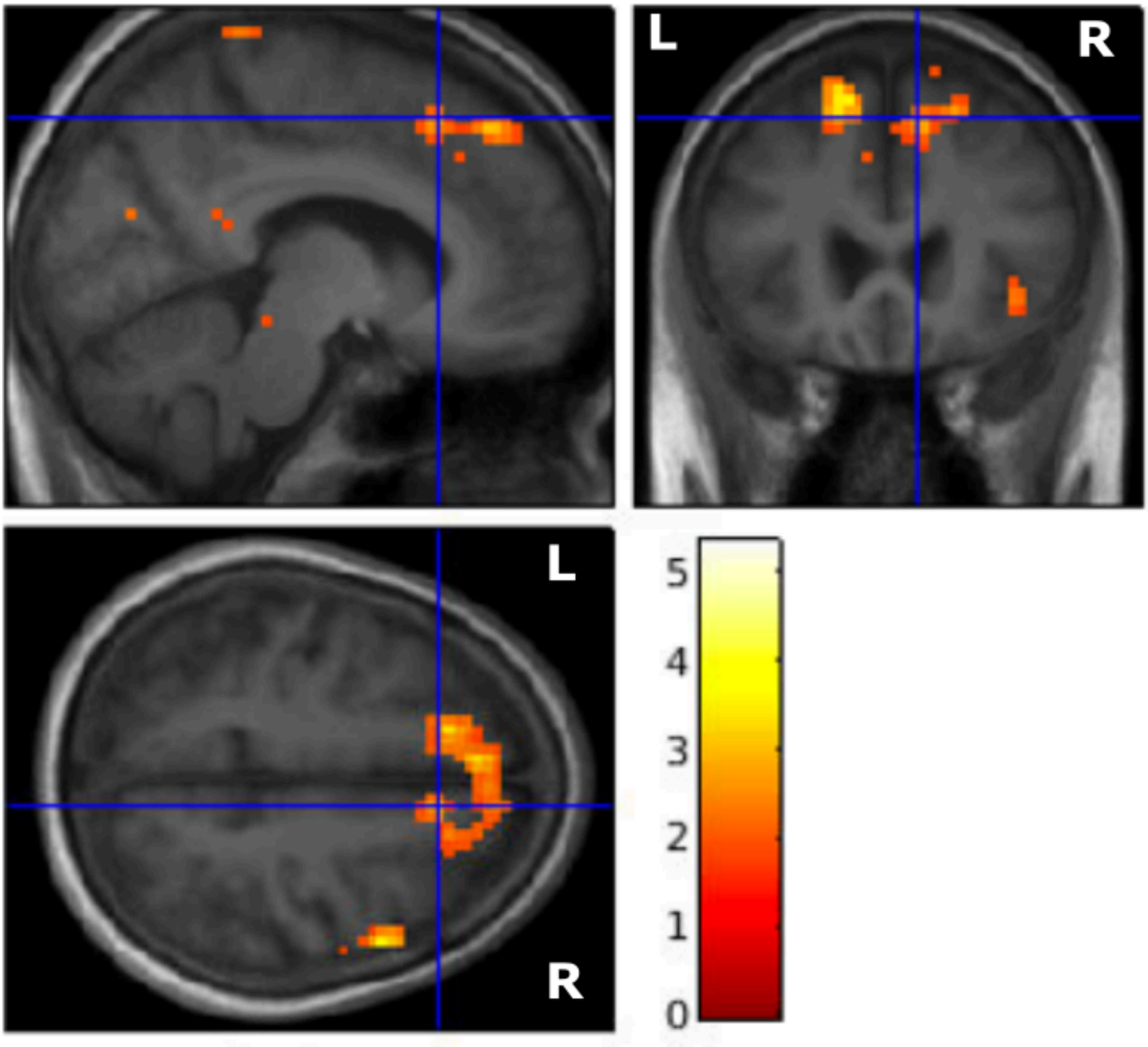

Supplementary Figure 1. SPM of the linear relationship between the anti-dyskinetic effect and the suppressive effect on no-go activity without the participant with the strongest negative TMS-induced modulation of preSMA activity and the strongest improvement in dyskinesia severity, thresholded at $p<0.05$ (uncorrected). MNI-coordinates x,y,z = 9,23,50 (same as figure 5 in main manuscript).
